# Supplementary material for: Rethinking infrastructure design from component failure to systemic resilience
Source: Nat Commun. 2025 Nov 3;16:9681. doi: 10.1038/s41467-025-64683-6 (PMC12583699; doi:10.1038/s41467-025-64683-6)
Supplement: Supplementary file 1 — Supplementary Information [file 41467_2025_64683_MOESM1_ESM.pdf]

Sam Dulin[1,2], Stergios-Aristoteles Mitoulis[3,4], Alexandre Bredikhin[5], Eric Treyz[6,1], Billy Leung[7], Jeffrey Dykes[7], Owen Karpeles[7], Shreeya Gurav[7], Alex Karhunen[7], Igor Linkov[1,8,*]

[1] Environmental Laboratory, US Army Engineer Research and Development Center, 696 Virginia Rd., Concord, MA 01742.

[2] Credere Associates, Westbrook, ME 04092.

[3] The Bartlett School of Sustainable Construction, University College London, 1-19 11 Torrington Pl, WC1E 7HB, UK.

[4] MetaInfrastructure.org, London, UK.

[5] U.S. Army Corps of Engineers, Pittsburgh, PA 15222.

[6] Bates College, 2 Andrews Rd, Lewiston, ME 04240.

[7] Regional Economic Models Inc., 433 West St, Amherst, MA 01002.

[8] Department of Engineering and Public Policy, Carnegie Mellon University, Wean Hall, 5000 Forbes Ave, Pittsburgh, PA 15213.

1 Supplementary Information

1.1 Historical Examples of Bridge Failure Ripple Effects

The recent collapse of the Francis Scott Key Bridge in Baltimore has brought renewed attention to the far-reaching consequences of infrastructure failures. The Key Bridge collapse, in which debris blocked the path of routes to the port and significantly impacted port operations and regional transportation networks, is not an isolated event. Rather, it is part of a historical pattern that underscores the complex interdependencies within modern infrastructure systems. While our analysis focuses on one recent example, there is a concerning historical trend taking place: degrading bridges that are places over navigable waterways. These examples emphasize the need for a systems-level approach to infrastructure resilience and disaster preparedness.

The Sunshine Skyway Bridge collapse in 1980 provides a stark example of how a single infrastructure failure can disrupt multiple systems simultaneously. When a freighter collided with the bridge during a storm, causing a significant portion to collapse into Tampa Bay, the immediate consequences extended far beyond the tragic loss of life. The fallen structure blocked the shipping channel, effectively shutting down maritime traffic to the Port of Tampa for several days [1]. This disruption not only affected local commerce but also rippled through regional and national supply chains that relied on the port's operations.

Similarly, the collapse of the Tacoma Narrows Bridge in 1940, while famous for its dramatic footage, is less often discussed in terms of its broader infrastructural impact. The bridge's failure, caused by wind-induced vibrations, severed a critical transportation link across the Tacoma Narrows. This disruption persisted for months, necessitating the development of alternative routes and significantly impacting local economies [2], [3]. The incident not only highlighted the vulnerability of large-scale infrastructure to environmental forces but also demonstrated how the loss of a single link could reverberate through an entire regional transportation network.

More recently, the Morandi Bridge collapse in Genoa, Italy, in 2018 offered a contemporary illustration of the intricate relationships between various infrastructure systems. The bridge, a crucial component of the city's highway network, also served as a vital link to the Port of Genoa, one of the Mediterranean's busiest ports. Its collapse not only disrupted road traffic but also significantly impacted port operations, underscoring the close interdependence between road infrastructure and maritime commerce [4]. The incident forced a reevaluation of infrastructure maintenance practices and highlighted the economic vulnerabilities created by aging transportation networks.

The 2002 collapse of the I-40 Bridge over the Arkansas River near Webbers Falls, Oklahoma, provides another compelling example of how bridge failures can have wide-ranging consequences. Beyond its immediate impact on road traffic, the collapsed structure blocked the McClellan-Kerr Arkansas River Navigation System, a critical inland waterway. This obstruction affected barge traffic and commerce along a significant portion of the river system, demonstrating how a localized infrastructure failure can disrupt a vast transportation network spanning multiple states [5].

These historical examples illustrate that bridge collapses and similar infrastructure failures can have complex, system-wide impacts that extend far beyond their immediate vicinity. The interconnected nature of modern infrastructure means that a single point of failure can lead to cascading effects across various sectors, including transportation, commerce, and regional economies. As such, it is crucial for researchers, policymakers, and engineers to adopt a holistic, systems-level approach when assessing infrastructure vulnerabilities and designing resilience strategies. Future research should focus on mapping these interdependencies more comprehensively and developing integrated approaches to mitigate the widespread impacts of potential infrastructure failures.

Furthermore, there is a number of bridges which are also at risk of blocking off ports and causing much wider repercussions potentially. Across the United States, numerous bridges play similarly crucial roles:

- The Verrazzano-Narrows Bridge in New York spans The Narrows, a critical access point for the Port of New York and New Jersey, the largest port on the East Coast [6].
- The Vincent Thomas Bridge in Los Angeles crosses the main channel of the Port of Los Angeles, one of the busiest container ports in the world [7].
- The Sunshine Skyway Bridge spans Tampa Bay, providing access to Port Tampa Bay, Florida's largest port by cargo tonnage [8].
- The Calcasieu River Bridge in Louisiana is critical for access to the Port of Lake Charles, a major petrochemical shipping hub [9].
- The Gerald Desmond Bridge in Long Beach, California, is a vital link for nearly 15% of the nation's waterborne cargo, connecting the Port of Long Beach to the mainland [10].
- The Blatnik Bridge, connecting Duluth, Minnesota, and Superior, Wisconsin, is essential for the Port of Duluth-Superior, the largest port on the Great Lakes [11].

These bridges, among others, represent potential single points of failure for significant portions of the U.S. maritime commerce infrastructure. A failure of any one of these could have far-reaching economic consequences, disrupting supply chains and impacting industries far beyond the immediate vicinity of the bridge.

While the bridges mentioned above are well-known and generally well-maintained, they represent only a fraction of the bridge infrastructure in the United States. According to the American Society of Civil Engineers' 2025 Infrastructure Report Card, 45% of all bridges are at least 50 years old, and 6.8% are considered structurally deficient and in poor condition [12]. This aging infrastructure presents a significant challenge, as the rate of deterioration is outpacing repair and replacement efforts.

What's particularly concerning is the possibility that the most at-risk bridges may not be the ones we're most aware of. Major bridges, such as the Verrazzano-Narrows bridge, like those listed earlier are subject to regular inspection and maintenance due to their obvious importance. However, smaller bridges over navigable waterways, particularly in rural or less economically vibrant areas, may be subject to less rigorous oversight despite their potential to cause significant disruptions if they fail. The true extent of the risk may be even greater than we realize. Many bridges that could potentially impact navigable waterways or critical infrastructure if they failed may not be adequately identified or monitored. The Federal Highway Administration somewhat tracks this issue, but a comprehensive inventory that considers the resilience of regions to collapses, rather than just risk, of all such bridges is a monumental task [13].

References

[1] S. Magazine, “Tampa bay sunshine skyway bridge disaster,” *Structure Magazine*, 2025. Accessed: 2025-08-22.

[2] H. Petroski, *To Engineer is Human: The Role of Failure in Successful Design*. Vintage, 2011.

[3] Washington State Department of Transportation, “Tacoma narrows bridge aftermath,” 2025. Accessed: 2025-08-22.

[4] Port of Genoa, “French commission visit genoa after morandi collapse,” 2025. Accessed: 202508-22.

[5] J. Styron, “May 26, 2002 webbers falls i-40 bridge collapse,” tech. rep., Oklahoma Department of Transportation, 2004.

[6] Port Authority of New York and New Jersey, “Port information,” 2025. Accessed: 2025-08-22.

[7] Port of Los Angeles, “Business overview,” 2025. Accessed: 2025-08-22.

[8] Port Tampa Bay, “About port tampa bay,” 2025. Accessed: 2025-08-22.

[9] Port of Lake Charles, “How the port became the nation’s top lng export hub,” 2025. Accessed: 2025-08-22.

[10] Federal Highway Administration, “Port of long beach - gerald desmond bridge replacement project,” 2020. Accessed: 2025-08-22.

[11] Duluth Seaway Port Authority, “Official website,” 2025. Accessed: 2025-08-22.

[12] American Society of Civil Engineers, “2025 infrastructure report card.” https://infrastructurereportcard.org/, 2025. Accessed: 2025-08-22.

[13] Federal Highway Administration, “National bridge inventory,” 2025. Accessed: 2025-08-22.
